# WRISK voices: A mixed-methods study of women's experiences of pregnancy-related public health advice and risk messages in the UK

**DOI:** 10.1016/j.midw.2022.103433

**Published:** 2022-10

**Authors:** Rebecca Blaylock, Heather Trickey, Julia Sanders, Clare Murphy

**Affiliations:** a Centre for Reproductive Research & Communication, British Pregnancy Advisory Service, 30-31 Furnival Street, London, United Kingdom; b School of Social Sciences, Cardiff University, United Kingdom; c School of Healthcare Sciences, Cardiff University, United Kingdom

**Keywords:** Pregnancy, Public health, Risk, Mixed-methods

## Abstract

**Background:**

Women receive many public health messages relating to pregnancy which are intended to improve outcomes for babies and mothers. However, negotiating the risk landscape and maternity care system can feel confusing and disempowering. Relationships between women and their healthcare providers are paramount, but they can be adversely affected by issues of trust and autonomy.

**Methods:**

We used a nested study design including an online survey and qualitative interviews to gain an understanding of women's experiences of risk messages during pregnancy. We purposively sampled survey participants to ensure the interview population included women whose voices are seldom heard and are disproportionately impacted by poor risk communication.

**Results:**

A total of 7,009 women responded to the survey, and 34 women participated in interviews. Participants received public health and risk messages from a range of sources. Data showed that women wanted a balance between a “better safe than sorry” approach and evidence-based information and advice. Women reported a discrepancy between the topics they received a lot of information on and areas in which they felt they needed more advice. Many participants said they were given conflicting advice, and the way information was delivered sometimes challenged their autonomy. We identified that younger women (<20 years old) and women with higher BMIs experienced stigmatisation in their maternity care.

**Conclusions:**

Our research shows the importance of risk communication that respects women's autonomy and trusts them to make decisions about their own pregnancy. We identified a need for a layered approach to risk communication. Whilst some women are happy to adopt precautionary behaviour without discussion, others will want a thorough examination of the evidence-base. Our findings suggest that more individualised care, continuity, and less judgement and stigmatisation from HCPs will improve experiences for women and may lead to better engagement with services.

## Introduction

Public health messages, or messages about risk regarding behaviours during pregnancy, form one aspect of a laudable agenda to improve pregnancy outcomes and give babies a better start in life during “the first 1000 days” (First 1000 days of life, 2019). These messages are based on an understanding that information and clear direction will lead expectant parents (primarily mothers) to make decisions that reduce the risk to the pregnancy, and of their babies experiencing short- and longer-term adverse health outcomes.

Pregnancy is often characterised as a ‘teachable moment’ during which women are receptive to the provision of public health messages and interventions (Antenatal care, 2021; Atkinson et al., 2016). One consequence is that women before and during pregnancy, are subjected to a wide range of messages about how to reduce or manage a multitude of risks. As part of routine antenatal care, midwives are expected to provide information on diet, smoking and substance misuse, weight management, emotional and mental health, exercise, pre-existing medical conditions, foetal movements, and pregnancy related symptoms, all in addition to providing clinical monitoring of the woman and her pregnancy (Sanders et al., 2016). Family members, friends, and parenting ’experts’ also contribute their own, sometimes contradictory, opinions and experiences. New study findings introduce an increasing range of risks and benefits – from avoiding air pollution (Smith et al., 2017) to eating more broccoli (Li et al., 2018). These findings are reported in press statements and media headlines, which do not always fully reflect the nuances of results, which may create alarm, or additional pressure for women and the people who care for them (Marshall et al., 2021).

The volume and intensity of instruction may be leading to unintended negative effects – including excessive maternal anxiety (Rowe and Fisher, 2015), and a failure to deliver personalised care that addresses individuals’ concerns (Sanders et al., 2016). Furthermore, risks may not always be communicated in a way that reflects or explains the limitations of the evidence base (Sharp et al., 2018).

Elements of current pregnancy-related public health advice have been challenged on the basis that they are insufficiently nuanced. For example, research which sought women's experiences of the UK Governments’ guidance to abstain from drinking alcohol in pregnancy, raised concerns about the communication of the precautionary principle (the ‘better safe than sorry’ approach) (Communicating public health alcohol guidance for expectant mothers 2018). Other concerns highlighted were the risk of irresolvable anxiety amongst women who had drunk alcohol *before* they knew they were pregnant and the encouragement of social surveillance of women's decisions. Alarm has also been raised about the extension of pregnancy risk messaging to all women of childbearing age, including the recent World Health Organization draft recommendation that all women with childbearing potential abstain from drinking alcohol (Global alcohol action plan 2022). Some suggest that the current risk messaging context may be driving a wider culture of parenting that tends to hold mothers responsible for any and all ills that befall their children (Lee and Bristow, 2020). In some countries, such as the USA, mothers are held maximally responsible and sometimes imprisoned for behaviours that may harm the foetus in utero such as taking illegal, and even prescription, drugs (Nešpor and Csémy, 2016).

We anticipated that the challenges in negotiating risk-messages disproportionately affect women who have pre-existing or pregnancy complications, or who are more vulnerable in other ways. Women who are taking medications, have higher BMIs, older or younger than the contemporaneous mean, or those living in poverty can all find themselves under pressure to change their behaviour whilst also finding risks more difficult to avoid. Mothers themselves, and those responsible for their care, sometimes struggle to balance maternal need for pharmacological treatments with limiting foetal exposure to (potentially) teratogenic medications (Lawthom, 2018). The MBBRACE-UK inquiries have identified advice to pregnant women to discontinue antidepressants as a contributory factor in maternal suicide, and there are concerns that restrictions on sodium valproate use amongst women of reproductive age may result in the deaths of pregnant women with epilepsy (Knight et al., 2021). A failure to prioritise maternal health can also have grave impacts for a woman's foetus. New research suggests that 5% of women with Hyperemesis Gravidarum who did not receive appropriate treatment had an abortion, and over half considered terminating otherwise wanted pregnancies (Nana et al., 2021).

We aimed to gain an in-depth understanding of women's lived experiences of pregnancy related risk messages and public health advice across a wide range of topics and issues.

### Aims

1) To describe women's lived experiences of risk communication relating to pregnancy in the United Kingdom and how this varies for:
  a. women with higher BMIs (>30 kg/m^2^)
  b. women who make decisions relating to medications, including for Hyperemesis Gravidarum, and for depression or anxiety
  c. younger women
2) To describe which aspects or topics relating to risk communication for pregnancy are problematic for women and why
3) To explore how women experience risk communication in terms of prioritising foetal and maternal health
4) To explore how women experience the communication of uncertainty and the precautionary principle as part of risk messaging

## Methods

### Study design

We used a mixed-methods nested design consisting of an online survey and in-depth interviews with a sub-sample of survey respondents.

### Study population

The study population consisted of women who were pregnant or who had been pregnant in the last five years. This included women whose pregnancy ended in a live birth, miscarriage, stillbirth, or termination of pregnancy.

*Inclusion criteria:*

• Women aged 16–45 years old
• Women who were or had been pregnant within the last 5 years
• Women who were living in the United Kingdom

*Exclusion criteria:*

• Women with insufficient English to participate in the online survey or interviews

The survey and interviews were inclusive of all people who had been pregnant in the last five years. We use the word *women* collectively throughout this paper as that is how most participants self-identify, but refer to individuals using their self-described gender. We use the term “BAME”, but acknowledge that this is problematic and present our data on ethnicity in more granular and specific terms in Table 1 of the Results (The Power of Language, 2021).

**Table 1:** Survey population characteristics.

	n (7090)	%	Mean
SOCIODEMOGRAPHICS			
*Age (years)*			
16–18	22	0.31	–
19–20	56	0.79	–
21–25	557	7.86	–
26–30	1850	26.09	–
31–35	2664	37.57	–
36–40	1540	21.72	–
41–45	354	4.99	–
45+	21	0.30	–
Missing	26	0.37	–
*Highest level of education*			
Secondary school	264	3.72	–
Apprenticeship/HND/NVQ	592	8.35	–
A-Levels	745	10.51	–
degree	2275	32.09	–
Postgraduate degree	1536	21.66	–
Missing	1678	23.67	–
*Relationship status*			
Single	129	1.82	–
Married	3663	51.66	–
Have a partner & live with them	1484	20.93	–
Have a partner	97	1.37	–
Divorced	11	0.16	–
Widowed	1	0.01	–
Separated	27	0.38	–
Other	38	0.54	–
Missing	1640	23.13	–
Ethnicity*			
White: English/Welsh/Scottish/Northern Irish/British	4898	69.08	
White: Irish	53	0.75	
White: Gypsy or Irish Traveller	1	0.07	
White: Other	299	4.22	
Mixed/multiple ethnic groups: White & Black Caribbean	24	0.34	
Mixed/multiple ethnic groups: White & Black African	12	0.17	
Mixed/multiple ethnic groups: White & Asian	34	0.48	
Asian/Asian British: Indian	23	0.32	
Asian/Asian British: Pakistani	5	0.07	
Asian/Asian British: Bangladeshi	1	0.01	
Asian/Asian British: Chinese	7	0.10	
Black/African/Caribbean/ Black British: African	2	0.03	
Black/African/Caribbean/ Black British: Caribbean	7	0.10	
Prefer not to say	46	0.65	
Other	43	0.61	
Missing	1635	23.06	
*Gender*§			
Female	5392	76.05	
Male	3	0.04	
Prefer not to say	17	0.24	
Other	20	0.28	
Missing	1658	23.39	
*Receive State benefits?*			
*Yes*	1165	16.43	
*No*	4560	64.32	
*Missing*	1365	19.25	
PREGNANCY HISTORY			
*Gravidity*		–	2.24
*Currently pregnant?*			
Yes	1348	19.01	–
No	5523	77.90	–
Missing	219	3.09	–
*Was most recent pregnancy planned?*			
Planned	5162	72.81	–
Unplanned	1208	17.04	–
Neither	475	6.70	–
Prefer not to say	26	0.37	–
Missing	219	3.09	–
*How did most recent pregnancy end?*			
Live baby	5328	75.15	–
Abortion	64	0.90	–
Miscarriage	163	2.30	–
Stillbirth	10	0.14	–
Neonatal death	6	0.08	–
Prefer not to say	17	0.24	–
Still pregnant	1283	18.10	–
Missing	219	3.09	–

⁎ We acknowledge the term “BAME” is problematic and present our data on ethnicity in more granular and specific terms in this table (The Power of Language 2021).

§ The project was inclusive of all people who had been pregnant in the last five years regardless of their gender identity. “Other” gender identities included non-binary and gender fluid.

### Data collection

#### Survey

The online survey was developed in collaboration with the project advisory group.. A pilot of the survey allowed the research team to draw on feedback from users and test dissemination strategies.

A participant information sheet and consent form were integrated into the survey. The survey included questions on participants’ experience of the advice, information, and support they received from different sources during pregnancy, with the focus on the respondent's most recent pregnancy. Questions with open free-text responses were included alongside questions with Likert scales and multiple-choice responses. Data on sociodemographic characteristics were collected. We also asked survey participants whether they would be prepared to be contacted for a future one-to-one interview.

The survey was hosted on SurveyMonkey (Surveymonkey, 2022), took approximately 10–15 min to complete, and was open between 12 June and 7 August 2019. There were 27 questions in total, although the final 8 questions were focused on participation in future research including the interviews. Data generated by the other 19 questions are presented in the results section.

The survey was disseminated through Facebook's inbuilt advertising feature which invited women aged 16–45 years from across the UK to participate, and through partner organisations that work with expectant and new parents. The survey link was shareable which allowed for snowball sampling (Johnson, 2014).

#### One-to-one interviews

To gain an in-depth understanding of women's experiences we also conducted interviews. A sampling frame was designed to ensure 20% of the interview sample were eligible for means-tested State benefits, at least 20% of the sample were from Black, Asian, and Minority Ethnic (BAME) backgrounds, and included at least six women with experience of pregnancy advice in relation to:

• having a higher BMI (>30)
• medications for mental health conditions
• medications for Hyperemesis Gravidarum
• being a younger mother (aged <20 at the time of their pregnancy)
• having a termination due to a perceived or actual risk either to themselves or their foetus/baby

Some participants fell into more than one of these categories. Survey participants that met the sampling criteria were selected to be contacted using a random number table, until the required numbers were obtained.

The participant information sheets (PIS), consent forms, and topic guides for the interviews were developed in collaboration with the project advisory group. Several topic guides were tailored to ensure they were suitable for participants who experienced different pregnancy outcomes (e.g., stillbirth, live birth, termination). Narrative topic guides allowed participants to tell their pregnancy stories in chronological order. Participants were asked to discuss their most recent pregnancy; however, many also drew on their wider experiences of pregnancy and parenthood.

Interviews (including pilot interviews) were carried out by two experienced female members of the research team (RB and HT) between April and November of 2019. Interviews lasted approximately 45–60 min. Participants were offered the option of in-person or telephone interviews. Two pilot interviews were conducted in-person and were deemed to be of a high enough quality to be included in the analysis. All subsequent interviews were conducted by telephone and recorded using a dictaphone. Participants received high street vouchers worth £20. Interviewers made detailed field notes following each interview. Audio files were transcribed verbatim using a commercial transcription service. Electronic transcripts were stored separately to any identifiable data on a secure IT system, and audio files deleted once they had been checked for accuracy.

### Ethical approval

Ethical approval was granted by the Research and Ethics committee of the School of Social Sciences at Cardiff University SREC/3201.

### Data analysis

Survey data were processed and analysed using Microsoft Excel and STATA SE 15 (StataCorp 2017).

The one-to-one interviews were analysed thematically following Braun and Clarke's method (Braun and Clarke, 2006). Transcripts were coded and analysed using Dedoose (SocioCultural Research, 2018). All transcripts and accompanying data such as researcher's field notes were read in detail several times by both interviewers, and high-level codes pertaining to the original research questions were identified. We identified further themes in the data during this inductive process, resulting in a coding framework informed by the data itself. Narrative summaries of data pertaining to each code were produced and cross-checked by two researchers, and subsequently organised into high-level themes.

The qualitative data from the interviews and quantitative data from the survey were ‘read’ alongside each other and are presented together (Ivankova et al., 2006).

### Research team

The research team is trained in and had extensive experience of working in pregnancy-related research and practice including social sciences (RB and HT), medical law (JS), public health (RB, JS, and HT) and clinical midwifery (JS). The research project was motivated and guided by the team's commitment to the values of feminist research (Jenkins et al., 2019) and informed choice.

### Patient and public involvement

We formed a project oversight group to provide input into our research design, data collection materials, and project outputs. This group included representatives from maternity user groups, advocates, healthcare professionals, and researchers.

## Results

A total of 7090 women responded to the survey and 34 took part in subsequent qualitative interviews. The qualitative data from the interviews and quantitative data are presented together. Main themes constructed through our inductive analysis were: sources of information and advice; better safe than sorry; information gaps; conflicting advice; challenges to autonomy; and stigmatised risk communication.

Sociodemographic information and pregnancy history for the survey population is presented in Table 1, and for the interview subpopulation in Table 2.

**Table 2:** Interview population characteristics.

	n (34)	%		Mean	Range
SOCIODEMOGRAPHICS					
*Age*					
16–18	0	0		–	–
19–20	2	5.88		–	–
21–25	9	26.47		–	–
26–30	7	20.59		–	–
31–35	7	20.59		–	–
36–40	7	20.59		–	–
41–45	0	0		–	–
45+	1	2.94		–	–
Missing	1	2.94		–	–
*Highest level of education*					
Secondary school	2	5.88		–	–
Apprenticeship/HND/NVQ	3	8.82		–	–
A-Levels	6	17.65		–	–
Undergraduate degree	13	38.24		–	–
Postgraduate degree	10	29.41		–	–
Missing	0	0		–	–
*Relationship status*					
Married	23	67.65			
Have a partner & live with them	9	26.47			
Have a partner & live separately	1	2.94			
Polyamorous	1	2.94			
*Ethnicity*					
White: English/Welsh/Scottish/Northern Irish/British	24	70.59			
Black/African/Caribbean/ Black British: African	3	8.82			
Mixed/multiple ethnic groups: White & Black African	1	2.94			
Asian/Asian British: Chinese	1	2.94			
Asian/Asian British: Indian	1	2.94			
Black/African/Caribbean/ Black British: Caribbean	1	2.94			
Mixed/multiple ethnic groups: White & Black Caribbean	2	5.88			
Mixed/multiple ethnic groups: White British & Middle Eastern	1	2.94			
*Gender*					
*Female*	33	97.06			
*Non-binary*	1	2.94			
*Receive State benefits?*					
Yes	10	29.41			
No	23	67.65			
Missing	1	2.94			
PREGNANCY HISTORY					
*Gravidity*	–	–		2	1 – 5
*Live births*	–	–		1.21	1 – 4
*Terminations/abortion*	–	–		0.26	0 – 2
*Miscarriage/stillbirth*	–	–		0.32	0 – 2

### Sources of information and advice

Participants received information about how to have a healthy pregnancy from a range of sources. In addition to their healthcare providers (HCPs), women sought out or received information from social media, the mainstream media, family and friends, often with contradictory messages.

#### Media

Several participants reflected on the emotional impact of reading “scaremongering stories” in the traditional media. One woman described how she had read an article about avoiding carrying heavy items during pregnancy and subsequently believed she had experienced a miscarriage because she had carried a car seat up a flight of stairs;

*“I remember reading somewhere you shouldn't be carrying heavy loads and things, this, that and the other…I think I was moving my daughter's car seat from my car and I carried it up a flight of stairs and then literally I think a day or two later that's when I had the miscarriage. I'm thinking maybe it was because of that, that's why it happened. (WRI4)*

Whilst many participants described, “panicking about everything” (WRI4), feeling “stressed and helpless” (WRI32), many also recognised that warnings about avoiding risks during pregnancy were “not 100% achievable” (WRI30) and you've “got to take it all with a pinch of salt” (WRI27).

#### Social media

The majority of interview participants spoke about social media being a source of helpful information and advice, and many had positive experiences of “due-date” Facebook groups. Similarly, participants who had conditions such as Hyperemesis Gravidarum found that social media provided peer support. However, many also recognised that social media was rife with misleading information and contradictory advice. Participants who were in transnational pregnancy support groups noted the contradiction in pregnancy-related advice across countries such as the number of routine antenatal check-ups offered to women.

#### Family and friends

Participants were overwhelmingly positive about the support provided to them by friends and their own mothers. In addition to providing practical support, they offered advice and reassurance in the face of an overwhelming landscape of pregnancy-related risk information, as was the case with WRI1 who described how her mum supported her when she was concerned about taking medication prescribed for Hyperemesis Gravidarum:

*“I think my mum was very supportive and saying, like, “If the doctors have prescribed it, it'll be fine and, like, you need it,” basically.” (WRI1)*

Partners were described by a small number of participants as being “helpful and supportive”. However, several participants expressed that they felt pressure to consider their partner's feelings when making decisions about their lifestyle during pregnancy. One woman described how her partner asked her to give up Coca Cola:

*“when…you've got someone else in your life perhaps who's panicking and, like, “Argh,” it's a little bit more pressure than you would want. You have to…tell them to calm down and rein it in a little bit. Like, “It's going to be okay, don't worry,” you know, “I'm still going to look after myself. I'm still going to look after this baby.” (WRI30)*

### Better safe than sorry

The survey data showed that women wanted a balance between a “better safe than sorry” approach and evidence-based information and advice (Table 4). The majority (67%; *n* = 4752) either ‘strongly’ or ‘tended’ to agree that when “scientists cannot rule out the possibility of harm pregnant women should take a better safe than sorry approach”, and 72% (*n* = 5106) wanted straightforward “do or don't” advice. There were some scenarios in which participants felt a particularly strong need to exercise caution and adopt the precautionary principle, including when making decisions around the consumption of alcohol and use of prescription medication:

*“Because no one really knows what's a safe amount and what isn't, and they are saying now, aren't they, just don't bother drinking anything because we don't know what is a safe amount. I've had maybe three drinks during pregnancy, and not even finished them. So, god, such a negligible amount. It's been absolutely fine. I've been alright with that, I've felt really good. And I think I needed that. I've just seen it as, like, a detox” (WRI32)*

**Table 4:** Advice during pregnancy and the precautionary principle (survey results).

	n (7090)	%
*I wanted straightforward “do/don't” advice to guide my decisions and protect the health of my baby*		
Strongly agree	2938	41.44
Tend to agree	2168	30.58
Tend to disagree	309	4.36
Strongly disagree	112	1.58
Prefer not to say	5	0.07
Missing	1558	21.97
*Some of the advice and information I received made me feel anxious that I might have harmed by baby*		
Strongly agree	714	10.07
Tend to agree	1823	25.71
Tend to disagree	1855	26.16
Strongly disagree	1129	15.92
Prefer not to say	11	0.16
Missing	1558	21.97
*I wanted to have all the available evidence before making decisions about my pregnancy*		
Strongly agree	3246	45.78
Tend to agree	1896	26.74
Tend to disagree	307	4.33
Strongly disagree	65	0.92
Prefer not to say	18	025
Missing	1558	21.97
*When scientists can't rule out the possibility of harm to the baby, women should be advised to take a ‘better safe than sorry’ approach*		
Strongly agree	2006	28.29
Tend to agree	2746	38.73
Tend to disagree	629	8.87
Strongly disagree	120	1.69
Prefer not to say	31	0.44
Missing	1558	21.97
*If a woman is not ready to stop drinking alcohol then she should use effective contraception*		
Strongly agree	2319	32.71
Tend to agree	1985	28.00
Tend to disagree	815	11.50
Strongly disagree	276	3.89
Prefer not to say	137	1.93
Missing	1558	21.97
*For me personally, I feel that if I had even one alcoholic drink when I was pregnant then that would be one drink too many*		
Strongly agree	1909	26.93
Tend to agree	1363	19.22
Tend to disagree	1439	20.30
Strongly disagree	801	11.30
Prefer not to say	20	0.28
Missing	1558	21.97

Despite the support for the precautionary principle, most survey respondents (73%; *n* = 5142) also wanted to have all the available evidence in order to make their own decisions about their pregnancy. Survey free-text responses and interview data showed that this extended to situations where there was a paucity of evidence or uncertainty:

*“A lot of standard advice seems to be based on a better safe than sorry mindset especially as it's not always possible to conduct high quality studies on pregnant women to generate robust evidence. I personally find it more useful to understand the evidence base and use this to make informed decisions, rather than be given a list of rules to follow. Every individual and every pregnancy is different and a do/don't approach fails to acknowledge this” (survey respondent)*

Some participants recognised that their own circumstances made them more cautious about certain decisions and behaviours. This was particularly the case with women who had experienced a previous pregnancy loss who exercised a greater deal of caution in subsequent pregnancies:

*“I put two and two together in that scenario and thought, “Yes, that's my fault.” … I can't change it. I just think, when it came to my next pregnancy, I changed my habits. I really, really, toned down my drinking, especially in the run up to getting pregnant. I think I waited, before I would have a glass of wine at weddings and stuff like that. I think I probably waited until I was well over the 12 weeks.” (WRI24)*

### Information gaps

In the interviews and survey, women reported a discrepancy between the topics they received a lot of information on and areas in which they felt they needed more advice. The following were the top four topics that participants said were well-explained: what to/not to eat during pregnancy (45%, *n* = 2484); vaccinations during pregnancy for influenza and whooping cough (44%, *n* = 2446); smoking before and during pregnancy (40%, *n* = 2211); and drinking alcohol before and during pregnancy (39%, *n* = 2148).

In contrast, participants wanted more advice and information on: managing mental health and pregnancy (40%, *n* = 2229); managing stress and pregnancy (32%, *n* = 1750); infections such as Group B Streptococcus, Toxoplasmosis, and Cytomegalovirus (30%, *n* = 1671); and infant feeding (30%, *n* = 1635). Many participants expressed that they received information that was not relevant to them at the expense of other areas where they needed better support:

*“Although I didn't smoke or drink I received much better information on this than anything else. I didn't receive any information on maintaining good mental health during pregnancy and I feel this contributed to me developing PND” (survey respondent)*

Furthermore, participants described feeling unable to act on the information about risk given to them. This was particularly the case with mental health conditions, and ‘risk factors’ which were unmodifiable at the time of pregnancy such as age or weight:

*“God, I couldn't tell you which journal this paper came out in but it was the study that looked at elevated cortisol in pregnant women and behavioural issues in their children… That really stuck with me because it came out and I was the most stressed person. I was so stressed and I was trying to do things to de-stress but life was just so all over the place that nothing worked.” (WRI32)*

### Conflicting advice

Many participants said they were given conflicting advice by HCPs on a range of issues including what to expect during labour and birth; diagnosis of pregnancy-related conditions; and the safety of alcohol consumption during pregnancy. A few women also noted that advice at the policy level had changed over the course of each of their pregnancies leading to confusion:

“I’*ve had four pregnancies in four years, so one in 2015, ‘16, ‘17, ‘18, and every single time the differences in just the protocols, the way things have changed, information and how you're given it. Every single time, it's been completely different.” (WRI21)*

Several participants described conflicting messages from HCPs about the safety of medication in pregnancy:

*“As soon as she [GP] saw that I was on medication for mental health, she was like, "So you're on antidepressants?" I said, "Yes." She went, "Well, what are we going to do about that?" I was like, "Well, my midwife said it's fine, the risk to my baby. The risk to myself if I came off them outweighed that, and it's a very minimal risk. I was on a low dose. She said, "It's not okay. It's not okay." She was very sharp with me. It's like as if I was taking hard drugs her something, her reaction was. She wouldn't give me a prescription for Citalopram. She said, "You need to go on Sertraline." I said, "I don't feel comfortable with changing meds at the moment." It took me a long time to find Citalopram and that really suits me. She was like, "But you can't breastfeed on Citalopram." She was assuming a lot of things, because I wasn't planning on breastfeeding anyway. I took the prescription from her for Sertraline and I was crying after the appointment. I went to the pharmacy thinking, "I'll just do it." Then the pharmacy were really funny with me because I was getting Citalopram and Sertraline, because I needed to wean myself off Citalopram first to start the Sertraline. That was humiliating because it was in front of everybody.” (WRI33)*

Such conflicting messages are not without consequences. In addition to the anxiety and distress caused, contradictory messages around the safety of medication can lead women to abstain from taking prescribed medication out of fear of harming their baby:

“I *am epileptic and though my seizures increased during pregnancy I was too scared to tell my midwife as I was afraid of my medication being increased and the risks to my unborn baby. I ended up being hospitalised during pregnancy because of my seizures and my daughter had to be delivered early by caesarean section. I believe if I had been given more information about the medication including risks and percentages that I wouldn't of been so scared to increase my medication.” (survey respondent)*

### Challenges to autonomy

The way in which information about risk was delivered to women sometimes challenged their autonomy and had negative implications for their relationships with their HCPs (Table 3). Many women initially implicitly trusted their HCPs. However, partial, or inaccurate advice from HCPs was a source of anxiety for women, and sometimes led to poor care:

“I'd aske*d several times, “Is there anything I can take for that?” and she'd said, “No, there's nothing we can give you. You just have to see it through.” it was a flat: “No, there is nothing you can take. There is no anti-sickness medication you can take in pregnancy. You just have to see it through. I just believed her. I thought, “Right, okay.” … I was worried that, if I did then go ask anybody else or try and take something, it wouldn't be safe for my baby. Like I say, she was really friendly. She was very personal… She built a good relationship up with you quickly, so I felt like, “No, she knows what she's doing. Trust her.” I was so unwell that I spent most of my days sleeping” (WRI19)*

**Table 3:** Judgement and blame during pregnancy (survey results).

	n (7090)	%
*I felt trusted to make my own decisions about what was best for me and my baby*		
Strongly agree	1823	25.71
Tend to agree	2509	35.39
Tend to disagree	854	12.05
Strongly disagree	330	4.65
Prefer not to say	16	0.23
Missing	1558	21.97
*I sometimes felt that my family and friends judged me for my choices or actions*		
Strongly agree	619	8.73
Tend to agree	1307	18.43
Tend to disagree	1492	21.04
Strongly disagree	2081	29.35
Prefer not to say	33	0.47
Missing	1558	21.97
*I sometimes felt that healthcare providers judged me for my choices or actions*		
Strongly agree	601	8.48
Tend to agree	1234	17.40
Tend to disagree	1890	26.66
Strongly disagree	1786	25.19
Prefer not to say	21	0.30
Missing	1558	21.97
*I sometimes felt that the general public judged me for my choices or actions*		
Strongly agree	502	7.08
Tend to agree	1281	18.07
Tend to disagree	1830	25.81
Strongly disagree	1880	26.52
Prefer not to say	39	0.55
Missing	1558	21.97
*I sometimes felt judged because of my age (old or young)*		
Strongly agree	424	5.98
Tend to agree	789	11.13
Tend to disagree	1628	22.96
Strongly disagree	2660	37.52
Prefer not to say	31	0.44
Missing	1558	21.97
*I sometimes felt judged because of my weight (over or underweight)*		
Strongly agree	759	10.71
Tend to agree	1082	15.26
Tend to disagree	1402	19.77
Strongly disagree	2253	31.78
Prefer not to say	36	0.51
Missing	1558	21.97
*Mothers are unfairly blamed for any negative outcomes in their babies and children*		
Strongly agree	1434	20.23
Tend to agree	2190	30.89
Tend to disagree	1515	21.37
Strongly disagree	280	3.95
Prefer not to say	113	1.59
Missing	1558	21.97

Several participants reflected that the use of carbon monoxide testing undermined their relationship with midwives who they felt were trying to ‘catch out’ women who underreported their smoking levels:

*“I always thought it's bizarre asking someone for their subjective answer and then you are almost like, “Right, well that means absolutely nothing because we need to do a test of the carbon monoxide in your blood anyway.” I think that's rubbish, really, because the relationship between midwife and mother is really, really important. I think we should be making sure that's as strong as possible throughout pregnancy….I think [testing] strengthens that power imbalance between clinician and patient” (WRI32)*

Several survey respondents also reported their data being shared with third parties, such as Slimming World, which further undermined trust between them and their midwife:

*“Because of my weight (high BMI) I was offered an additional service about losing weight which I did not want, I declined and it still was pushed on me and she turned up to my appointment without my consent” (survey respondent)*

A breakdown in trust has serious implications for women's relationships with their midwives and other care givers. Although 61% (*n* = 4332) of survey participants reported feeling trusted to decide what was best for themselves and their babies, 26% (*n* = 1835) reported that they sometimes felt judged by HCPs for their choices and actions. Some described lying to their midwives about their behaviours because they felt judged:

“I gene*rally felt I was judged for some of the foods I ate and the occasional alcoholic drink. As a pharmacist, I used my professional knowledge to make judgements even though this went against the advice of the midwife. As a result I often did not tell or lied to the midwife about my choices.” (survey respondent)*

### Stigmatised communication of risk

We identified that younger women (<20 years old) and women with higher BMIs had a distinct and stigmatised experience of maternity care.

#### Younger mothers

Women who became mothers under the age of 20 had a distinct experience of risk messaging, and several described the advice they received as instructional. One woman explained that she “felt like if I didn't do something, with my first baby especially, that they'd take my baby off me or something” (WRI31). One interview participant (WRI9) with four children said that the advice she received during her pregnancies changed as she got older. During her first pregnancy she found the support from a health visitor to be useful but “condescending”. In contrast, during her later pregnancies she found HCPs to be more respectful of her wishes.

One woman (WRI11) suggested that family and friends compensated for a lack of information provided about labour and birth by HCPs, and was concerned that women even younger than herself may find the paucity of advice and information “scary”. WRI9 suggested that all first-time mothers should be treated the same, regardless of their age:

*“I think they should … be treated like any other first-time mum, because I bet there are 30-odd-year-old mums out there that probably know the same amount as an 18-year-old who's pregnant for the first time. If you've not done it before, you don't know.” (WRI9)*

Another younger participant reflected on how partial advice left them unprepared for the consequences of obstetric injuries:

*“I wish I'd known more about obviously tears and stuff. … it was the sort of situation where they said about it, but they sort of went, “Oh, you're young, you won't have to worry about that sort of thing. Third degree tears normally happen to older ladies,”” (WRI3)*

#### Women with higher BMIs

Approximately 26% (*n* = 1841) of survey respondents reported feeling judged because of their weight and described dehumanisation and depersonalisation within the maternity care system. This is exemplified by one woman who was present for conversations about how staff would move her should she become incapacitated in labour:

“I acc*ept that there is also the risk of the idea of my body as an object which is large. Therefore, in itself, poses a risk to staff who are handling my body. That is a very depersonalising way to think about oneself I'm aware that is something that has to be considered, but I feel quite strongly that risk needs to be considered in private and not in front of me.” (WRI22)*

Another described how her weight dominated every interaction with HCPs, leading her to feel like her pregnancy would not end with a healthy baby:

*“My weight dominated every conversation held with every single medical professional and led me and my partner to be absolutely convinced we wouldn't bring the baby to term. We were told repeatedly that the birth would be difficult, I wouldn't cope and that there would be complications. I was told to have an epidural at the first signs of labour as I would def need emergency intervention so it made sense to do it at the beginning as I wouldn't be able to control myself enough once the labour had started to do it later on…. I was almost convinced I wouldn't be bringing my baby home and even didn't do things like put up a* cot*” (survey respondent)*

Participants also described a failure to recognise efforts they had made in the preconception period to mitigate against risk:

*“I was overweight and many of my appointments seemed to focus more on my weight than anything else despite the fact that I proved I had lost more than 6 stone in the 18 months before falling pregnant” (survey respondent);*

and a failure to contextualise risk:

*“As I'm overweight I felt very anxious all the way through the pregnancy as I was constantly being told I was at a higher risk but no one could tell me by how much. It regularly made me feel like I wasn't good enough to have a baby” (survey respondent)*

## Discussion

Overall, participants wanted a balance between a ‘better safe than sorry’ approach and evidence-based information and advice. Whilst most women were happy to adopt the precautionary principle, others wanted more detailed information relating to risk. A need for a well-informed layered approach to information provision was evident to support honest discussions about risk, including where there was uncertainty.

Other research on pregnancy related COVID –19 public health messages found that women felt it was better to be “*safer* than sorry”, and over-interpreted advice to stringently socially-distance, “shielding” from the outside world (Sanders and Blaylock, 2021). Reflecting the hesitancy amongst pregnant women to accept the COVID-19 vaccination (Skirrow et al., 2022; Blakeway et al., 2022), in this research we found that even when counselled on the importance of using medication by HCPs, some women preferred not to do so out of fear of harming their baby, leaving them vulnerable to serious deterioration in their health . Whilst the adoption of the precautionary principle may be considered effective public health messaging, we found concerning examples that the precautionary approach enacted through self-policing behaviours can lead to negative outcomes for the woman herself. We found that different women had different appetites for risk, and this was often shaped by previous experiences. Whilst cognisant of the fact that there was unlikely to be a causative link between a behaviour such as carrying something heavy or drinking a small amount of alcohol and miscarriage, some women having experienced miscarriage felt that no avoidable risk was acceptable when it came to subsequent pregnancies. However, this was not universal, and what may have been a small or negligent risk to one person (e.g., drinking a small amount of alcohol) may have felt like a huge risk to another.

Women have different appetites for risk, with their tolerance often changing over time shaped by their own experiences. In the absence of any evidence of harm about a particular behaviour, they should be free to adopt or reject the precautionary principle (McDonald et al., 2011). However, it becomes more challenging where there is a known risk to the fetus such as in the prescribing of drugs with teratogenic effects such as Sodium Valproate (Valproate Pregnancy Prevention Programme 2022). Whilst being well motivated, formal healthcare guidelines that mandate a precautionary approach undermine women's autonomy, are stigmatising, and remove their ability to make decisions for themselves (Royal College of Midwives 2021; Lee et al., 2021). Increasingly, women who make what others deem to be ‘risky’ decisions are seen as transgressive according to social norms which dictate what an ideal mother should do (Kukla, 2008).

Our participants explained that there was a mismatch between the topics they received a lot of information on and areas in which they felt they needed more advice and support. For example, smoking and drinking alcohol in pregnancy were identified as topics which participants received a lot of information. In contrast, participants wanted more advice on managing mental health conditions during pregnancy. Some participants felt that this over-focus on certain topics caused them to experience bad outcomes, such as post-natal depression. Gaps in the information provided to pregnant women, and conflicting advice, may leave them to make their own decisions without any support. Without any guidance from healthcare professionals on issues such as whether it is safe to take particular medications in pregnancy, they are left to the mercy of the sea in which they swim- the prevailing cultural ideas about what is and is not appropriate behaviour in pregnancy and the imperative to protect the foetus at all costs- even at their own expense (Maternity Decisions Induction Survey, 2021).

The attention given to some topics in public health messaging during pregnancy arguably reflects the research agenda and accessibility of data. There is a trend particularly within the Developmental Origins of Health and Disease (DOHaD) paradigm, to use large cohort studies to ascertain associations between maternal exposures and foetal outcomes. There is an implicit assumption about the “causal primacy of maternal pregnancy effects” and this sets the agenda of DOHaD research which is reinforced and reproduced, rather than challenged (Sharp et al., 2018). Our research on the reporting of pregnancy related studies found that the majority of studies that were reported in the UK mainstream media frame mothers, rather than protective towards their unborn infants, as vectors of potential harm to their children, who are the focus of the health outcomes (Marshall et al., 2021). A recent report found an imbalance between women's research priorities, such as perinatal mental health and research funding, suggesting a realignment of research priorities with women's needs is urgently needed (Guthrie et al., 2020).

The use of carbon monoxide testing to validate women's self-reported behaviours, and referrals to additional support without consent were described as undermining the relationship between women and their midwives. Others have also identified the use of carbon monoxide testing in maternity care as having the potential to “do more harm than good” (Bowden, 2019). By not trusting women's self-reports, testing for carbon monoxide exposure leaves women feeling judged and not trusted which could lead to disengagement with services, and is at odds with shared-decision making (O'Brien et al., 2021). We found that when they felt judged, women were more likely to lie or hide their behaviour, which is consistent with findings from other research on working class mothers and smoking in Wales (Grant et al., 2020).

Women with higher BMIs and younger mothers reported routine stigmatisation, dehumanisation, and a lack of kindness within maternity care. Younger women described feeling they risked having their baby taken into care if they did not comply with instructions. To receive care under such a climate of fear hinders the formation of trusting relationships between women and their health care providers (Sanders et al., 2016; McLeish and Redshaw, 2019). Furthermore, around a quarter of women felt judged for their weight. As mentioned previously, when women feel judged they are less likely to engage in healthcare which can have ramifications for maternal and foetal health.

Women with higher BMIs described the distress caused by having to repeat their story multiple times, and a failure to recognise the weight-loss journey they may have been on prior to each appointment/meeting each new midwife. Within expanding midwifery continuity of carer (MCoC) teams, midwives should be able to provide more individualised care.

Some of our participants described being told they were “at risk” or had a high-risk pregnancy but not being told how much greater their risk of a poor outcome was. This was particularly the case with risk factors which were unmodifiable at the time of pregnancy such as having a high BMI or suffering from a pre-existing health condition. This failure to contextualise risk derives from how risk is calculated from population-level epidemiological studies and extrapolated and applied to the individual in a clinic setting (WRISK 2021).

Pregnancy is often characterised as a “teachable moment” during which healthcare professionals can educate women and improve their health (Atkinson et al., 2016). This leads to a greater focus on the individual determinants of health, rather than the social or structural determinants of health which require a longer lead-time and collective, population-level efforts to resolve. This focus on the individual determinants of public health is a departure from current trends within public health more broadly which focus on social determinants. Acknowledging the difficulty in trying to solve public health ‘problems’ in a 9-month window, there is a growing focus on improving women's health in the pre-conception period, regardless of their pregnancy planning intentions (Budds, 2021).

Whilst we did not interview HCPs, we identified that system-level constraints also contribute to the poor communication of risk and public health messages. The sheer volume of public health initiatives and information provided on a wide range of topics means that antenatal care appointments can become “tick-box” exercises, rather than tailoring the appointment to meet a woman's individual needs. Midwives are constrained by short appointment times, a lack of training, and resources (Sanders et al., 2016) which inevitably means that some information is prioritised. This is compounded by serious staff shortages (NHS, 2021). Again, MCoC may contribute to more individualised care but plans for universal implementation have been delayed (Sandall et al., 2016; Delivering Midwifery Continuity 2021).

These structural issues mean the quality of a woman's antenatal experience is contingent on who they happen to see that particular day, how knowledgeable they are, and whether they have time to meet their individual needs. This is evidenced by our findings that a woman with Hyperemesis Gravidarum was told by her midwife that there was no safe medication that she could take. More recently, anecdotal evidence emerged of pregnant women being turned away from COVID-19 vaccination centres or being told by their midwives that vaccination for COVID-19 is not safe in pregnancy- contrary to the evidence-base (The Guardian 2022). Ultimately, we are yet to decide who gets to choose what is an acceptable risk to take in pregnancy. Women's and HCPs’ appetites for risk might be different, and when they are discordant this can lead to gatekeeping and conflicting advice.

### Strengths and limitations

Our study included over 7000 women from across the UK. Our purposive sampling frame ensured our interview population included higher proportions of ethnic minorities and women on low incomes than at the UK population level, ensuring the most marginalised women were represented. However, the sampling frame design may have shaped our findings and focused attention on certain experiences of risk communication at the expense of others, for example, the stigmatisation of women with higher BMIs.

Our survey was self-selecting and may reflect the views of those more motivated to participate in research. Given the survey was hosted online, we are mindful that it excluded those without access to the internet. Furthermore, our survey and interviews were restricted to those who could speak English and therefore excluded non-English speakers who are likely to have more difficulties accessing and navigating the maternity care system in the UK.

## Conclusion

Our research shows the importance of risk communication that respects women's autonomy and trusts them to make decisions about their own pregnancy. Our findings support principles which have been previously identified in aiding high quality risk communication, and we recommend that HCPs involved in delivering risk messages familiarise themselves with these (Freeman, 2019). We identified a need for a layered approach to risk communication. Whilst some women are happy to adopt precautionary behaviour without discussion, others will want a thorough examination of the evidence-base and its limitations. Our findings suggest that more individualised care, continuity, and less judgement and stigmatisation from HCPs will improve experiences for women and may lead to better engagement with services. A realignment of research priorities with women's own needs will likely lead to improved evidence to support their care. Ultimately, our research demonstrates that women want what is best for their pregnancy and children. Women should be well informed and then trusted and supported to make decisions based on their own circumstances.

## Funding statement

This work was supported by the 10.13039/100010269Wellcome Trust as part of the WRISK Project [212089/Z/18/Z].

## CRediT authorship contribution statement

**Rebecca Blaylock:** Conceptualization, Data curation, Formal analysis, Investigation, Methodology, Project administration, Writing – original draft. **Heather Trickey:** Conceptualization, Formal analysis, Funding acquisition, Investigation, Methodology, Project administration, Supervision, Writing – original draft. **Julia Sanders:** Project administration, Supervision, Writing – review & editing. **Clare Murphy:** Conceptualization, Funding acquisition, Project administration, Supervision, Writing – review & editing.

## Declaration of Competing Interest

The authors declare that they have no known competing financial interests or personal relationships that could have appeared to influence the work reported in this paper.
